# Design of a Tablet Computer App for Facilitation of a Molecular Blood Culture Test in Clinical Microbiology and Preliminary Usability Evaluation

**DOI:** 10.2196/mhealth.5041

**Published:** 2016-03-18

**Authors:** Lasse L Samson, Louise Pape-Haugaard, Michelle C Meltzer, Martin Fuchs, Henrik C Schønheyder, Ole Hejlesen

**Affiliations:** ^1^Department of Health Science and TechnologyAalborg UniversityAalborgDenmark; ^2^AdvanDx Inc.Woburn, MAUnited States; ^3^Department of Clinical MicrobiologyAalborg University HospitalAalborgDenmark; ^4^Department of Clinical MedicineAalborg UniversityAalborgDenmark

**Keywords:** usability, mobile applications, tablet computers, clinical simulation, health information systems, diagnostic test, clinical microbiology

## Abstract

**Background:**

User mobility is an important aspect of the development of clinical information systems for health care professionals. Mobile phones and tablet computers have obtained widespread use by health care professionals, offering an opportunity for supporting the access to patient information through specialized applications (apps) while supporting the mobility of the users. The use of apps for mobile phones and tablet computers may support workflow of complex tasks, for example, molecular-based diagnostic tests in clinical microbiology. Multiplex Blood Culture Test (MuxBCT) is a molecular-based diagnostic test used for rapid identification of pathogens in positive blood cultures. To facilitate the workflow of the MuxBCT, a specialized tablet computer app was developed as an accessory to the diagnostic test. The app aims to reduce the complexity of the test by step-by-step guidance of microscopy and to assist users in reaching an exact bacterial or fungal diagnosis based on blood specimen observations and controls. Additionally, the app allows for entry of test results, and communication thereof to the laboratory information system (LIS).

**Objective:**

The objective of the study was to describe the design considerations of the MuxBCT app and the results of a preliminary usability evaluation.

**Methods:**

The MuxBCT tablet app was developed and set up for use in a clinical microbiology laboratory. A near-live simulation study was conducted in the clinical microbiology laboratory to evaluate the usability of the MuxBCT app. The study was designed to achieve a high degree of realism as participants carried out a scenario representing the context of use for the MuxBCT app. As the MuxBCT was under development, the scenario involved the use of molecular blood culture tests similar to the MuxBCT for identification of microorganisms from positive blood culture samples. The study participants were observed, and their interactions with the app were recorded. After the study, the participants were debriefed to clarify observations.

**Results:**

Four medical laboratory technicians, for example, representative of end users of the app, participated in the clinical simulation study. Using the MuxBCT app, the study participants successfully identified and reported all microorganisms from the positive blood cultures examined. Three of the four participants reported that they found the app useful, while one study participant reported that she would prefer to make notes on paper and later enter them into the LIS.

**Conclusions:**

The preliminary usability evaluation results indicate that use of the MuxBCT tablet app can facilitate the workflow of the MuxBCT diagnostic test.

##  Introduction

### Health Care Workers and Mobile Technology

Support of user mobility is an important aspect to consider when developing clinical information systems (CIS) [1-3]. Physicians and nurses continuously move between offices, wards, patients, and workstations while conducting their work. Other employees such as laboratory workers are also mobile in their work, as they move between labs, specialized equipment, and offices to acquire and report laboratory results [4]. With the ongoing trend of increased digitization of information in health care and thus a move away from paper documentation, this sets a requirement for clinical staff being able to access CIS while away from workstations.

In recent years, mobile devices in the form of mobile phones and tablet computers have become almost ubiquitous in daily life and their use has spread to clinical settings [5-7]. The mobile devices are generally lightweight, intuitive to use, and contain powerful data processing abilities. Thus, they offer a potential solution for providing the necessary access to CIS, while supporting the mobility requirements of health care professionals. While mobile phones and tablet computers are seeing more widespread use by health care professionals in clinical settings, there is still a need for more research to discover and document the benefits and potential difficulties these mobile devices may bring to particular applications (app) [7,8].

Mobile phones and tablet computers offer an interesting option for providing workflow assistance and guidance of complex tasks. For example, in clinical laboratories the development of specialized apps may facilitate the workflow of preparing, interpreting, and reporting results of diagnostic tests. In clinical microbiology, many new diagnostic tests have been developed in recent years that allow for rapid identification of microorganisms that cause bloodstream infections [9]. Many of these tests are based on molecular biology techniques and have the potential for providing more specific results in a shorter time than current tests. However, the molecular tests are often more complex to use compared to the conventional techniques and therefore may require more skill from the operators [10].

### Multiplex Blood Culture Test

Multiplex Blood Culture Test (MuxBCT) (AdvanDx, Woburn, MA, USA) is a new blood culture diagnostic test under development based on the molecular technique fluorescence in situ hybridization (FISH). The MuxBCT uses fluorescence-labeled, peptide nucleic acid (PNA) probes that bind to specific DNA targets in microorganisms, which can then be detected by fluorescence microscopy. Like other molecular diagnostic tests, the MuxBCT involves more complexity than conventional methods used for blood culture diagnostics in clinical microbiology. To reduce the time required for data capture, test interpretation, and results reporting, a MuxBCT tablet computer app was developed as a research project. The aim of the app is to reduce the complexity of the diagnostic test by guiding the user, for example, a medical laboratory technician (MLT), stepwise through the diagnostic test and the interpretation process of the MuxBCT diagnostic test. Additionally, the app will allow users to report results directly to the Laboratory Information System (LIS).

In this study, we describe the system design and development considerations for the MuxBCT tablet app, and we report the results of a preliminary usability evaluation of the MuxBCT app.

### The Multiplex Blood Culture Test App

#### App Description

The MuxBCT app has been developed as a research project and functions as an accessory tool to facilitate the use of the MuxBCT diagnostic test. The app was developed based on requirements set through observations of the workflow during blood culture analysis in a clinical microbiological laboratory [4]. End users have been involved in the development life cycle of the MuxBCT app, for example, by identifying flaws in a prototype of the app through a participatory heuristic evaluation [11]. The app is integrated with the LIS in use at the Department of Clinical Microbiology (DCM) at Aalborg University Hospital, Denmark. The LIS integration of the app is important for clinical use, as a lack of an integration would require reentry of test results and likely lead to an inefficient and more error-prone workflow [12]. The MuxBCT app is developed as a native app for the Android operating system and has been designed to support 10.1-inch tablet computers.

**Figure 1:**
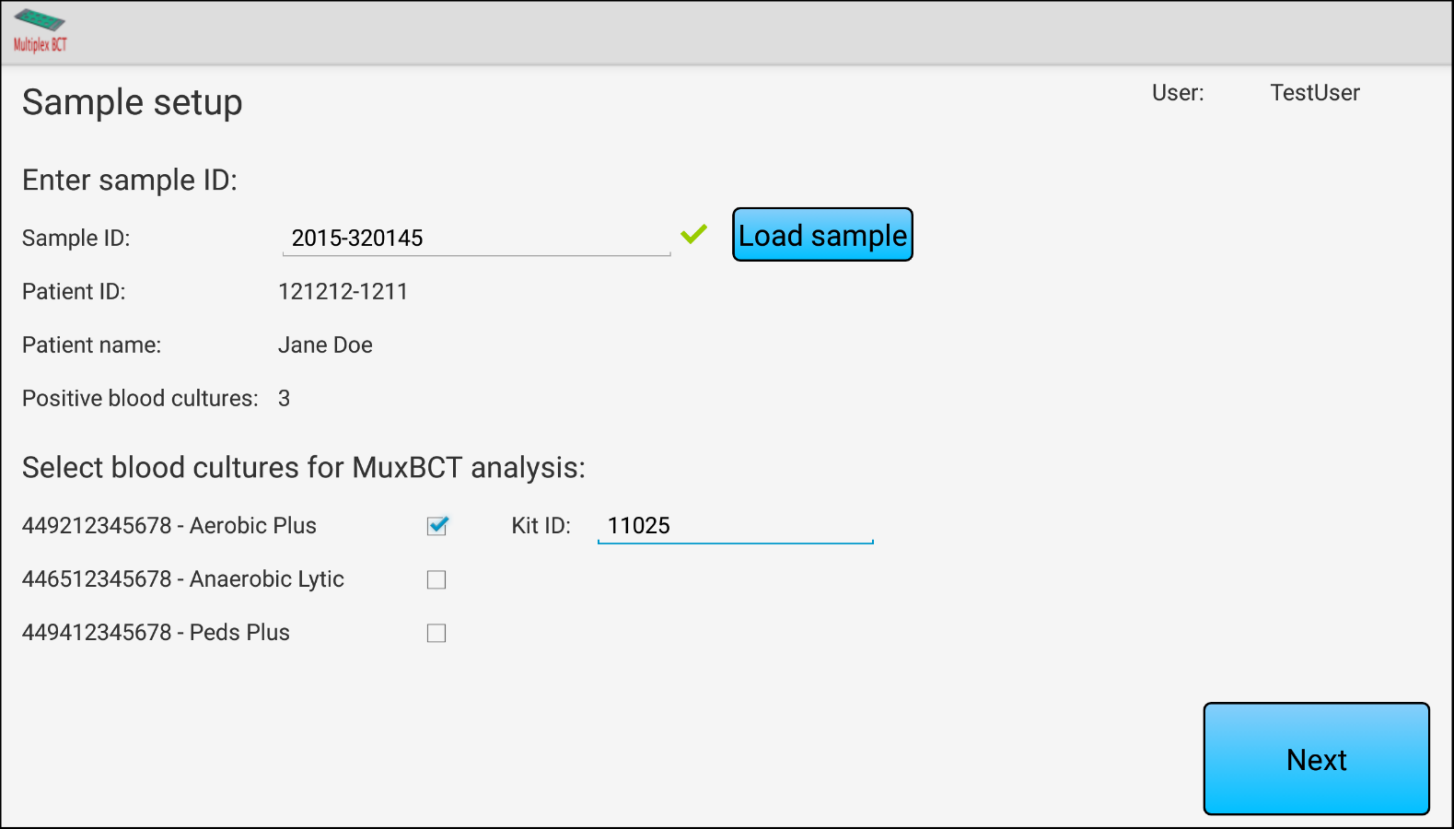
Sample screenshot of the Multiplex Blood Culture Test (MuxBCT) app showing the initial sample setup where a sample ID is entered into the system, data are retrieved from the Laboratory Information System (LIS), and displayed in the app. The patient information displayed in the image is fictitious.

When using the app, users must initially complete a sample setup (Figure 1 shows this), where the sample identification number is entered into the app. The sample identification number can optionally be entered via a barcode reader. The entered sample identification number is used to retrieve relevant clinical information about the patient and that sample from the LIS, which is then displayed to the user. The user selects one or more positive blood cultures for analysis. The Kit ID (lot information) for each MuxBCT diagnostic test used must be entered for each selected blood culture. The lot information is stored for quality control purposes.

**Figure 2:**
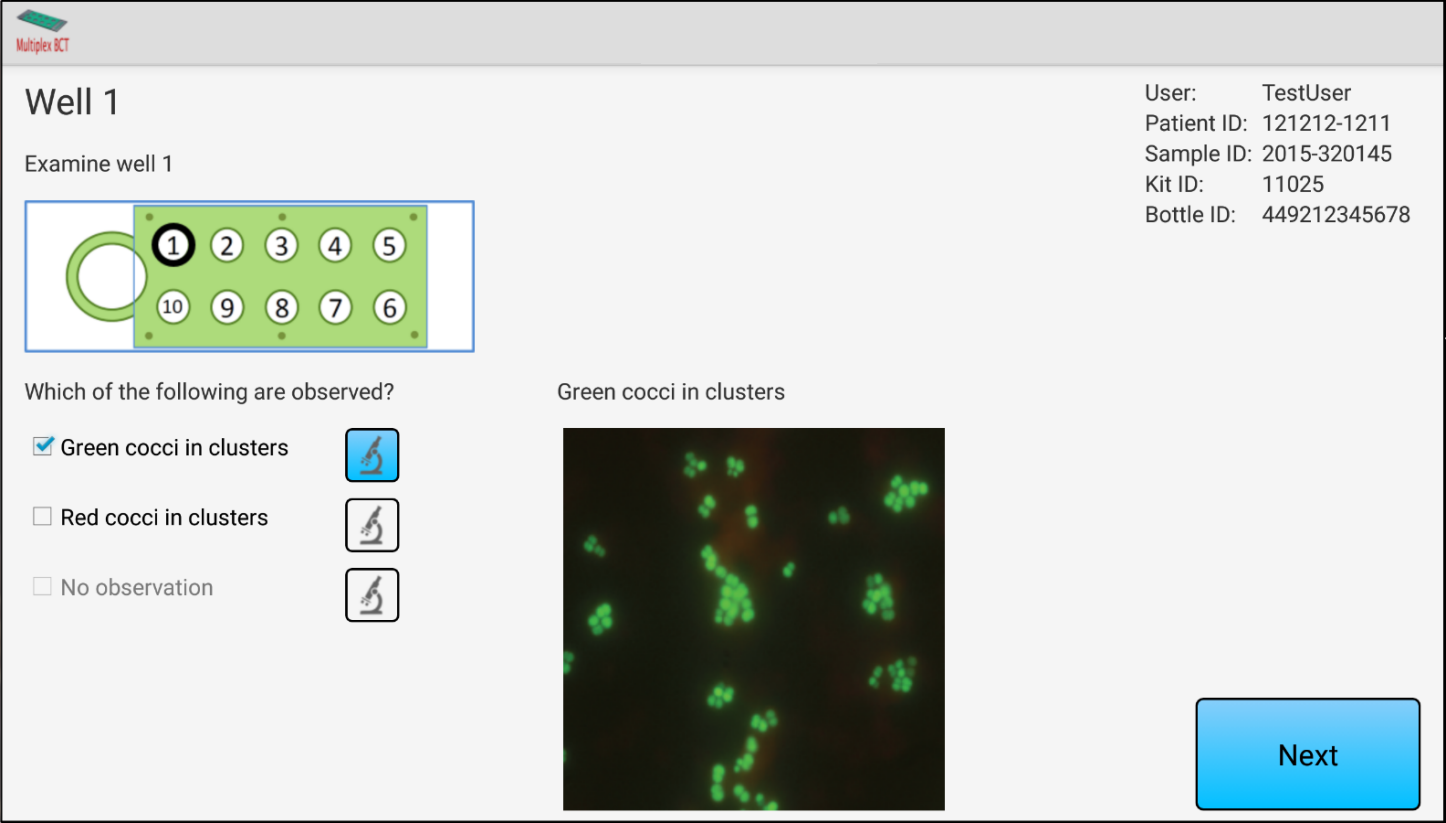
Sample screenshot of the Multiplex Blood Culture Test (MuxBCT) app showing data entry of test results acquired through fluorescence microscopy of the MuxBCT diagnostic test. The patient information displayed in the image is fictitious.

The MuxBCT diagnostic test is divided into 10 different analysis areas (wells). In most cases, a classification well followed by three identification wells will need to be examined. The user will start fluorescence microscopy with a classification well and will be prompted to enter the findings into the app through the use of multiple choice questions. The app has an algorithm that guides users to analyze only relevant wells based on observations entered for the classification well. During analysis of the selected identification wells (Figure 2 shows this), the user will again enter observations via multiple choice questions. In the user interface, users can choose to display reference images of the same type of fluorescent microorganism to assist them in analyzing their observations.

When the user has completed the test analysis, a result summary of any microorganism findings, for example, a bacterium finding and a yeast finding, will be displayed to the user. Once the user has submitted the analysis result, it becomes available for all relevant staff through the LIS. The user also has an option to enter notes along with the result, which will either be visible for all clinical staff or only staff in the clinical microbiology laboratory. Until the result is submitted to the LIS, users can go back in the app and revise any entered data.

The app offers a training mode where fictitious data are generated and displayed to the user. In the training mode, no data are communicated between the LIS and the tablet app. The training mode allows users to become familiar with the app before they use it to facilitate the analysis of positive blood culture samples.

#### System Architecture

**Figure 3:**
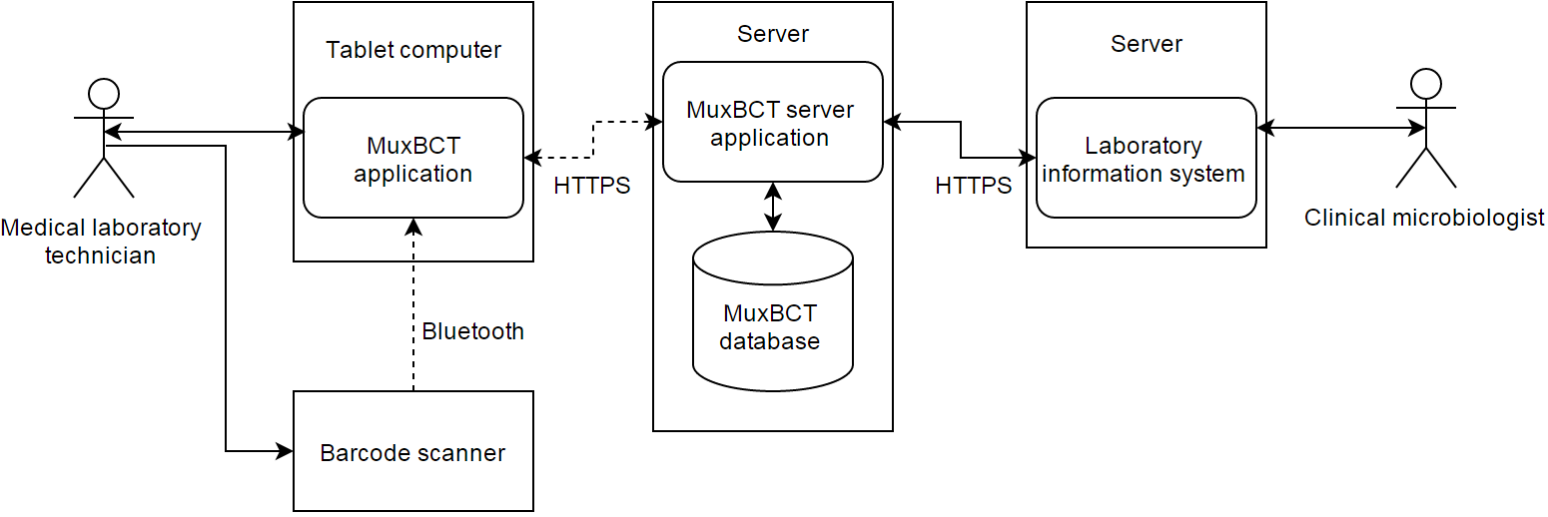
Overview of the Multiplex Blood Culture Test (MuxBCT) system design. The medical laboratory technician (MLT) will use the MuxBCT app for facilitation of the MuxBCT diagnostic test. The MLT can use a wireless barcode scanner to enter barcodes into the tablet app. Clinical microbiologists can access the MuxBCT results through the LIS. The HTTPS protocol is used to ensure encrypted communication of data. The arrowheads indicate direction of data flow. Dashed lines in the figure indicate wireless connections.

The MuxBCT system consists not only of a tablet computer with the MuxBCT app, but also of a server with a MuxBCT server app (Figure 3 shows this). The MuxBCT server communicates with the LIS of the clinical microbiology laboratory. Additionally, the tablet app supports the use of a Bluetooth barcode scanner as an accessory for the MuxBCT app to reduce the potential for data entry errors [13]. Once the scanner is connected, it allows for data entry by scanning sample identification barcodes. The MLTs interact directly with the MuxBCT tablet app during analysis of a MuxBCT diagnostic test.

The app exchanges data with a MuxBCT server, which is integrated with the LIS. The server processes any results received by the tablet app, so that the data can be exchanged with the LIS. The server app communicates with the LIS to receive patient information relevant to the sample being analyzed and to store results of the MuxBCT diagnostic test in the LIS. Once data have been transmitted to the LIS, they become available for clinical microbiologists, who can access it through the LIS. The clinical microbiologists can then communicate the results to a patient’s treating physician to optimize the patient’s treatment. When the DCM has approved the results in the LIS, the results become available from the electronic health records system through LIS integration.

A database is associated with the MuxBCT server, which is used to store test results. This allows data to be reviewed for quality assurance purposes. Additionally, user interactions with the system are logged in the MuxBCT database.

#### System Security

**Figure 4:**
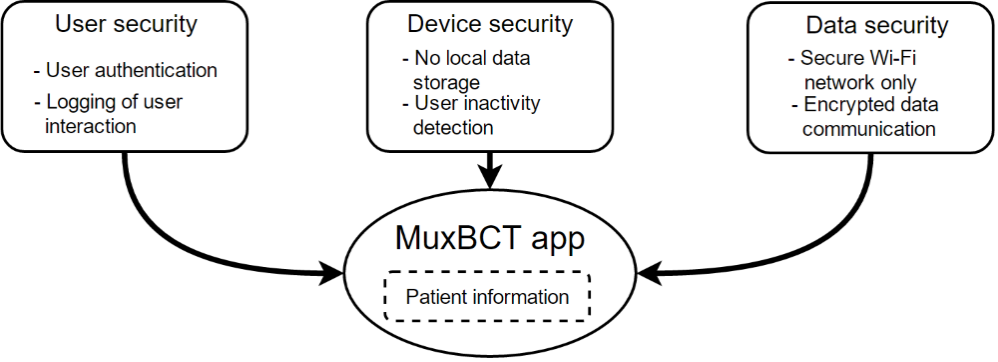
Overview of the security design considerations that in unison protect the patient information of the Multiplex Blood Culture Test (MuxBCT) application (app). It is a requisite that the security considerations are implemented before the deployment of the MuxBCT app. Wi-Fi is the local wireless network.

Because of an inherent risk of tablet computers more easily being misplaced or stolen than regular workstation computers, several security design considerations have been built into the MuxBCT app, which aims to keep patient data secure and private (Figure 4 shows this). The security design considerations are divided into three categories: user security, device security, and data security.

The user security design considerations consist of user authentication and of logging user interactions. To gain access to the functionality of the MuxBCT app, a user must provide a username and password, which must then be successfully authenticated. Additionally, any user interaction that provides access to patient information will be saved as a log entry in the MuxBCT server’s database. This enables an audit functionality, where any potential misuse of the system can be tracked, and users can be held accountable for their actions.

The device security is based upon not storing any data locally on the tablet computer and automatically logging out inactive users. To prevent potential patient data leaks, the app is designed to store data only temporarily in the memory of the tablet computer while the MuxBCT app is being used. Once a user completes a task with the app, any information related to that task is cleared from the tablet computer. If any users are inactive for more than a predefined period, for example, 20 minutes, the system will automatically log the user out of the system. The user will then have to authenticate again if she wishes to resume using the MuxBCT app. The system administrator can adjust the inactivity period that must pass before a user is logged out of the system.

Letting the tablet app exchange data with the MuxBCT server only through the hospital’s secure Wi-Fi network ensures data security. Additionally, all data communication is protected with encryption that relies on the HTTPS protocol (Figure 3). By encrypting the Bluetooth connection, data entered using an accessory Bluetooth barcode scanner are protected.

## Methods

### Study Design

To gain insights about the use of the MuxBCT app, a preliminary usability evaluation was conducted at the DCM at Aalborg University Hospital. This allowed the app to be tested in its normal context of use. The evaluation was structured as a clinical simulation study and aimed for a high degree of realism. The study took place within normal working hours at the DCM, which meant that normal minor background disturbances occurred, which further aided in making the study a near-live simulation for the participants. A scenario of use with a focus on realistic tasks was developed in cooperation with staff at the DCM, where the MuxBCT app was used to support result interpretation and data entry of FISH-based blood culture diagnostic tests. A pilot study was conducted with one MLT to validate the study design before the main study took place.

The study aimed to recruit 4-5 participants, which is commonly used as a cost-efficient approach for finding usability problems through usability evaluation methods [14]. An informational flyer about the study was presented to the MLTs of the DCM. For inclusion in the study, participants were required to have prior experience with blood culture analysis. Potential participants were excluded if they had any prior experience with MuxBCT or the MuxBCT app prototype. A senior staff member of the DCM handled selection of participants for the study, with a goal of achieving a mixed level of seniority among the participants. The participants were compensated for their time.

At the onset of each study session, the participants were introduced to the concept of the MuxBCT diagnostic test and the accessory tablet app. The first author acted as test leader during the study and provided the users with instruction and training. The study participants then participated individually in a MuxBCT app training session lasting roughly 10 minutes. The training session followed a structured approach so each participant had the same introduction to the app. If the study participant was not familiar with tablet computers and the Android operating system, they were given a brief introduction to this. Once the participant was comfortable with the tablet, a scenario of use of the MuxBCT app was described for the participant. Each participant carried out 5 predefined tasks using the MuxBCT app. The 5 tasks were divided into 24 steps, for which they received instructions to complete one at a time. If the participant was stuck on a step, they were allowed to ask questions and to receive help from the test leader.

During the study, the participants were not offered any assistance in the interpretation of diagnostic test results or in the use of the app. The participants were asked to think-aloud during the study. After the study, the participants were debriefed in a short interview that followed a semistructured approach, which was based on the observations made by the test leader during the study.

The test leader acted as an observer during the study and took notes of the participants’ interactions with the tablet app and the diagnostic tests. Furthermore, all interactions with the tablet app were recorded as a video file with the Android app Recordable, where user interactions with the app, for example, touch gestures, were saved as an overlay on the video. Audio was recorded using a dictation machine and by the built-in microphone of the tablet computer. The video and audio files were transferred to a computer and synchronized, allowing for analysis of the study results.

The debriefing interviews and the audio data from the users’ interaction with the app were analyzed by coding the data through the process of meaning condensation and categorization followed by an interpretation of the processed data [15]. An inductive approach was used in the analysis of video data, which was supported by the audio data of users thinking aloud during their interaction with the app. Different types of events were coded in categories based on their characteristics. Once structured into themes and categories, the data were summarized, which allowed for an analysis of the total time spent for MuxBCT analysis. In addition, the structured data were interpreted, which allowed for identifying the users’ perspectives about potential difficulties when interacting with the app.

### Materials

The MuxBCT diagnostic test was undergoing development at the time of the study. While the general concepts of the test were fully designed, it was not ready for use in a clinical microbiology laboratory. Therefore, the MuxBCT was unavailable at the time of the study. Instead, MuxBCT was simulated by using the QuickFISH Enterococcus and QuickFISH Candida (AdvanDx, Woburn, MA, USA) tests. The QuickFISH tests are predecessors to the MuxBCT and require the same fluorescence microscopy analysis to interpret test results, but each QuickFISH test identifies fewer microorganisms. By using the QuickFISH tests, two out of ten analysis wells of the MuxBCT were simulated. The use of the two QuickFISH tests necessitated that the study participants changed the microscopy slide once during the analysis phase. As part of the training session, it was explained to the participants that the QuickFISH tests were used to simulate the MuxBCT.

A senior MLT who had been involved in the MuxBCT app development process assisted in the study by helping with the preparation of the QuickFISH tests. Additionally, the senior MLT inoculated samples drawn aseptically from blood culture bottles negative at termination of incubation (BacT/Alert, bioMérieux, Marcy l’Etoil, France) with the bacteria, *Enterococcus faecalis* or *Enterococcus faecium*, and the yeasts, *Candida albicans* or *Candida glabrata*, so that each sample grew two of these microorganisms. Each study participant was assigned a random blood culture sample to analyze using the QuickFISH test kits. The study participants were kept blinded to the microorganisms in their assigned blood culture sample.

For the study, the MuxBCT app was installed on a Samsung Tab Pro 10.1-inch tablet computer (Samsung Electronics, Seoul, Korea) running Android 4.4.2. All text in the app was in Danish, the first language of the study participants. The tablet was connected to a CHS 7DiRx 1D Bluetooth barcode scanner (Socket Mobile, Newark, CA, USA) and the hospital’s secure Wi-Fi network. Access to the secure Wi-Fi network was approved by the hospital’s Information Technology Department before the study took place.

In preparation for the study, the MuxBCT app was connected to the MuxBCT server. However, because the study was a simulation, the MuxBCT server was not connected to the LIS during the study, but instead returned realistic, fictitious patient data to the tablet app. All MuxBCT analysis results were sent to the server and saved in the associated database for later validation.

## Results

### Study Participants

There were four MLTs that participated in the clinical simulation study, which was conducted in one day at the DCM. All study participants were female. The mean age of the study participants was 37 years, and the mean experience in blood culture diagnostics was 7 years (range 3-14 years). There were three participants that described their skill level with fluorescence microscopy as practiced, while one described her skill level as novice. All participants described themselves as experienced in the use of mobile phones and tablet computers based on personal use outside the clinical microbiology department. The study spanned across several rooms in the clinical microbiology department. Preparation of the QuickFISH molecular tests took place in one room with specialized equipment primarily for molecular diagnostics. The interpretation of the tests required fluorescence microscopy and took place in a dark room that was located near the first room. Training and debriefing took place in a larger room in the department, where there was little activity and few potential disturbances.

### Participant Results

All participants correctly identified all microorganisms in the simulated blood culture samples. The participants correctly entered their findings into the MuxBCT app and submitted them to the MuxBCT server. The analysis results were retrieved from the database of the MuxBCT server to verify that they had been transmitted successfully. It was confirmed that all results were stored correctly in the database. On average, the analysis of the blood culture sample and data entry of the results into the MuxBCT app took 10.2 min (range 6.5-16.0 min).

All results were entered correctly on the first attempt. However, when two participants attempted to mark their findings, they initially changed the reference image being displayed instead; this was the result of clicking on the microscope icon associated with each possible finding instead of marking the checkboxes corresponding to their findings (Figure 4). Both participants noticed that they had not entered the results and marked their findings successfully before continuing.

During the debriefing interview, three participants expressed that they found the system useful. A participant noted that she would prefer to continue making notes of analysis results on paper and entering them later into the LIS at a workstation. The main reasoning behind this was that the text on the tablet computer was too small to read during data entry (Figure 4). Another participant also expressed an interest in larger text on the app. Both of these participants wore glasses, which they would remove during use of the fluorescence microscope.

It was observed for all participants that they made only minor use of the reference images displayed during data entry. When prompted about this during the debriefing, two participants explained that the reference images were not needed during the study, as the samples being analyzed by fluorescence microscopy provided a clear picture. They remarked that the reference images would be useful in cases where the fluorescence signal was less clear. A participant noted that the reference images would likely be most valuable for users with limited experience in fluorescence microscopy.

All participants used the barcode scanner successfully to enter sample identification data into the app. A participant initially had troubles entering the sample identification with the barcode scanner because the relevant input field had not been selected before the barcode was scanned (Figure 4). Throughout the study, the Bluetooth barcode scanner functioned without loss of connection to the tablet computer. There were three participants that expressed that the mobility of the tablet in combination with the barcode scanner was satisfactory; however, one participant suggested that a slightly smaller tablet computer could be used.

The majority of the app’s security features were enabled and used successfully during the study. The user authentication was not enabled, as study participants logged in with fictitious user data. Additionally, the user inactivity detection was not enabled in the app, as the study participants were not involved in normal job functions during the study, and thus there was no risk of inactivity for long periods. During the study, there were no issues with loss of connection to the Wi-Fi network at the DCM.

## Discussion

### Principal Results

The MuxBCT tablet app was developed as a research project for the facilitation of the MuxBCT diagnostic test that is designed for rapid identification of bacterial and fungal pathogens in positive blood cultures. This study describes the MuxBCT app and a preliminary usability evaluation of the MuxBCT tablet app based on a clinical simulation study conducted at a clinical microbiology laboratory. The MuxBCT diagnostic test is intended to provide fast and accurate identification of microorganisms causing bloodstream infections, allowing the physician to treat with more appropriate antimicrobials sooner. However, the use of molecular diagnostic tests such as MuxBCT involves a more complex workflow than conventionally used methods of analysis [10]. Specialized apps such as the MuxBCT app may facilitate the workflow of diagnostic tests in the clinical laboratory, which can lead to a more accurate and faster result. In the case of treating patients with severe sepsis, this is critical, as each hour of delay in the administration of an effective antimicrobial treatment is associated with a decrease in survival rate [16]. While some specialized apps may be more suitable for use on existing workstation PCs, which are commonly distributed throughout clinical laboratories, this is not a practical solution for the MuxBCT app, as MuxBCT requires use in a dark room due to the use of fluorescence microscopy for reading the test results. As the app is designed to guide users through the interpretation process in a stepwise manner, the app needs to be accessible directly from the location where the test is being analyzed. The results of this study indicate that although there were some usability issues for the users to interact with the tablet in the dark room, the app was viewed as a useful adjunct to this kind of test and that the light from the tablet did not disturb the analysis process.

All participants in the study managed to correctly identify and report the microorganisms from the blood culture samples they examined. The foundation for the use of the app was a short training session. The participants were able to use the MuxBCT app for guidance during the examination of the QuickFISH molecular diagnostic tests. The QuickFISH tests with the same detection method were used to simulate the MuxBCT, which was still undergoing development at the time of the study. The initial classification well could not be simulated by use of QuickFISH tests in a realistic manner, so, as a compromise, the participant received instructions of what findings to input for this well. The classifier is a molecular equivalent to the classical Gram stain with minor differences in distinction between bacterial groups. Common use of the MuxBCT would require analysis of the classifier well followed by analysis of three identification wells. The analysis of additional identification wells is only necessary when the results from the classification well indicate a polymicrobial bloodstream infection.

The participants were able to access the necessary clinical information they required during the test without having to access the LIS using a workstation. Additionally, all participants successfully made use of the barcode scanner during the study. During the debriefing interviews, three out of four participants expressed that they found the system useful. A participant expressed a preference for writing the results on paper, for example, like the normal workflow during blood culture analysis, and later entering the results into the LIS. Some usability issues were noted during the study, but overall the usability evaluation found that the MuxBCT app was a reliable tool for facilitating the workflow of the MuxBCT diagnostic test, as all participants managed to correctly interpret, enter, and communicate the test results.

The results of the study have led to some updates of the MuxBCT app. The main structure of the app has not been changed; however, several changes have been made to improve the user interface of the app. For example, the font size has been increased throughout the app, making text more easily readable, and the check boxes where users mark their findings of microorganisms have been increased in size.

Based on the results of the development process and this study, we believe that the involvement of clinical microbiology staff in the development and evaluation processes of the MuxBCT app has led to an improved quality of the app. In general, there is a growing focus on the need for involvement of medical professionals throughout the entire app development process to ensure the quality of the app and to prevent possible patient harm [17].

The structure of the study was a near-live clinical simulation that aimed for a high degree of realism. The study was conducted in a clinical microbiology laboratory using simulated blood culture samples, molecular diagnostic tests, and fluorescence microscopy. The combination of these factors allowed the MLTs in the study to carry out their analysis with minimal assistance from the test leader. This provided findings about the usability of the system, which may not have been discovered if other usability evaluation methods had been applied. The use of a think-aloud protocol may have detracted from the realism of the study. However, this did not seem to cause any issues during the study. Furthermore, the think aloud-protocol was necessary to gain knowledge of the fluorescence microscopy observations and to confirm that the participants entered their observations correctly. The realism of the study may have been further diminished by a senior MLT assisting with the preparation of the QuickFISH tests and by the test leader observing the participants during the study, but this did not affect the context of use for the MuxBCT tablet app. Therefore, it is unlikely that it had any large impact on the results of usability evaluation.

A challenge in selecting a device for the MuxBCT app was to ensure mobility of the system, while providing enough screen space for displaying the necessary information clearly and allowing for easy input of data. The mobility of the system was tested as the study spanned several rooms in the clinical microbiology laboratory. Although the app has been designed for use on a 10.1-inch tablet computer, two of the study participants noted that they would prefer text size in the app to be increased. In regards to the mobility of the system, three study participants noted that they found the mobility of the system adequate, while one study participant would prefer a smaller tablet computer. It may be possible to redesign the app to support a slightly smaller tablet computer, while increasing the text size. However, this requires a careful redesign of the graphical user interface.

While the MuxBCT app was not connected to the LIS during this study, it was connected to the MuxBCT server. This meant that it was possible to test communication of test results and to validate that they were transferred correctly. To simulate LIS connectivity, fictitious patient data were returned by the MuxBCT server. The data were visible by the participant within the app during the study, which helped to ensure a high degree of realism. If the system had been connected to the LIS, it may have induced some additional delays during use of the app while data was being transferred. However, any such delays would only affect test setup and result reporting and would not have changed the evaluation of how the MuxBCT app aided in the analysis and interpretation of test results, as these steps of the MuxBCT app do not require communication with the LIS.

Data security of patient information is an important factor to consider in the development of apps for mobile phones and tablets [18,19]. To secure the privacy of patient information, several security related features were built into the MuxBCT app. The design of security features for the MuxBCT app has a high similarity to those described by Landman et al used in the implementation of the CliniCam mobile phone app [19]. While the MuxBCT app required access to the hospital’s protected Wi-Fi network for use, the participants did not experience any loss of connectivity during the study. As clinical laboratories are of fixed dimensions, it should be a manageable task to setup adequate Wi-Fi coverage throughout an entire laboratory. Other mobile device apps for facilitation of clinical workflow may experience challenges with sufficient Wi-Fi network coverage, as larger areas need to be covered [18,19].

Future development on the MuxBCT app could include the functionality to directly communicate test results electronically to selective stakeholders, for example, the patient’s treating physician or to clinical microbiologists within the DCM. This could potentially reduce errors related to communication of test results and reduce the total test turnaround time [20,21]. Future evaluation studies will be required to see how the MuxBCT app affects the use of the MuxBCT diagnostic test once both are put into daily use in a clinical microbiology laboratory. It should be stressed that the current iteration of the MuxBCT app was developed as a research project. If future development of the MuxBCT app aims to prepare it for commercialization and regular use in a clinical microbiology laboratory, the app must conform to national regulatory requirements governing medical devices.

### Limitations

A significant limitation of this study is the low number of study participants. Each participant, who was an expert in the field of use for the MuxBCT app, conducted an analysis of a positive blood culture sample with two microorganisms. This allowed for a total of 8 microorganisms being successfully identified. While this number is too low to accurately determine the quality of MuxBCT diagnostic test results being facilitated by the MuxBCT app, these results in combination with the results from observations and debriefing interviews provided an insight into the usability of the MuxBCT app.

The realism of the study was also diminished to some degree by the MuxBCT diagnostic test being unavailable for the study. The test was substituted with the QuickFISH tests, and as there is a high coherence between the QuickFISH tests and the MuxBCT, it is unlikely that this had a significant impact on the study results.

The results of the clinical simulation cannot be generalized, as they are specific to the MuxBCT app being evaluated. However, our study results indicate that a clinical simulation is a valuable tool for evaluating the usability of a mobile device app that aim to support clinical workflow. Other developers and implementers may find this as an effective tool to use preimplementation. The system design of the MuxBCT app and the considerations of security features may inspire the design choices of other mobile apps being developed for clinical use. However, their effect should be evaluated through a full system implementation in daily clinical workflow.

### Related Work

To our knowledge, there has not been any previous publication describing the use of a mobile phone or tablet computer app for facilitating the use of a diagnostic test in a clinical laboratory. However, the use of mobile phone or tablet computer apps designed for use by health care professionals in clinical settings is becoming increasingly common [7,8,18,19,22].

There is a growing interest in clinical simulation studies as a tool for evaluating usability of hospital information technology (HIT) [23,24]. It is a common goal of simulation studies to create a near-live simulation, which requires a setting representative to where the system will be used [1,23,25,26]. While most clinical simulation studies focus on the evaluation of stationary HIT, some simulation studies have been used to examine the usability of HIT designed for use on mobile devices [27,28]. This study differentiates itself from previous clinical simulation studies, as it took place in a clinical laboratory. This meant that the context of use for the system being evaluated did not involve patient contact, but involved a focus on the diagnostic test laboratory analysis process, which included a focus on the use of laboratory equipment as part of the user workflow. A realistic simulation of the use of specialized laboratory equipment may be difficult to achieve outside an actual clinical laboratory.

### Conclusions

The MuxBCT tablet computer app facilitates the workflow of a novel molecular diagnostic test using PNA FISH technology for blood culture analysis and was developed with system flexibility and security of patient data as high priorities. The app was developed based on previous analysis of user requirements. However, additional functionalities such as user authentication and user inactivity detection were added to create a secure app. The security features are vital to protect patient data. A preliminary usability evaluation was conducted as a clinical simulation study in a clinical microbiology laboratory. The study aimed to provide a high degree of realism by having end users conduct fluorescence microscopy of molecular diagnostic tests employing available PNA FISH technology, while using the MuxBCT app to facilitate the workflow. The study results indicate that users can successfully use the app to facilitate the MuxBCT diagnostic test workflow. All diagnostic test results were interpreted, entered, and communicated successfully to the MuxBCT server with use of the MuxBCT tablet app, which validates the system design considerations. The study provided useful feedback on the app usability, for example, in regards to font size and tablet size. The clinical simulation method can be a valuable tool for evaluating the usability of mobile apps intended for clinical use, as the method provides insights into complex use case scenarios where context of use is important.

## Abbreviations

app: application
CIS: clinical information system
DCM: Department of Clinical Microbiology
FISH: fluorescence in situ hybridization
HIT: health information technology
LIS: laboratory information system
MLT: medical laboratory technician
MuxBCT: multiplex blood culture test
PNA: peptide nucleic acid
